# Understanding Health Behavior Technology Engagement: Pathway to Measuring Digital Behavior Change Interventions

**DOI:** 10.2196/14052

**Published:** 2019-10-10

**Authors:** Heather Cole-Lewis, Nnamdi Ezeanochie, Jennifer Turgiss

**Affiliations:** 1 Johnson and Johnson Health and Wellness Solutions, Inc New Brunswick, NJ United States

**Keywords:** engagement, user engagement, health behavior, health determinants, digital behavior change intervention, measurements

## Abstract

Researchers and practitioners of digital behavior change interventions (DBCI) use varying and, often, incongruent definitions of the term “engagement,” thus leading to a lack of precision in DBCI measurement and evaluation. The objective of this paper is to propose discrete definitions for various types of user engagement and to explain why precision in the measurement of these engagement types is integral to ensuring the intervention is effective for health behavior modulation. Additionally, this paper presents a framework and practical steps for how engagement can be measured in practice and used to inform DBCI design and evaluation. The key purpose of a DBCI is to influence change in a target health behavior of a user, which may ultimately improve a health outcome. Using available literature and practice-based knowledge of DBCI, the framework conceptualizes two primary categories of engagement that must be measured in DBCI. The categories are health behavior engagement, referred to as “Big E,” and DBCI engagement, referred to as “Little e.” DBCI engagement is further bifurcated into two subclasses: (1) user interactions with features of the intervention designed to encourage frequency of use (ie, simple login, games, and social interactions) and make the user experience appealing, and (2) user interactions with behavior change intervention components (ie, behavior change techniques), which influence determinants of health behavior and subsequently influence health behavior. Achievement of Big E in an intervention delivered via digital means is contingent upon Little e. If users do not interact with DBCI features and enjoy the user experience, exposure to behavior change intervention components will be limited and less likely to influence the behavioral determinants that lead to health behavior engagement (Big E). Big E is also dependent upon the quality and relevance of the behavior change intervention components within the solution. Therefore, the combination of user interactions and behavior change intervention components creates Little e, which is, in turn, designed to improve Big E. The proposed framework includes a model to support measurement of DBCI that describes categories of engagement and details how features of Little e produce Big E. This framework can be applied to DBCI to support various health behaviors and outcomes and can be utilized to identify gaps in intervention efficacy and effectiveness.

## Introduction

### Background

Globally, the creative integration of technology solutions to address health issues is growing [1]. This integration has been largely through the promotive uptake of healthy behaviors to achieve desired health outcomes via technology [2]. In this paper, these technologies will be referred to as a digital behavior change intervention (DBCI). Recent systematic reviews demonstrate that digital interventions supported by behavior science evidence hold the key to driving critical behavior change processes that lead to improved health behaviors and health outcomes [2]. Common examples of targeted behaviors include smoking cessation, increase in physical activity, improvements in dietary habits, medication adherence, and safe sexual practices [3].

However, the extent to which these innovations can deliver on the promise of demonstrable positive health outcomes depends on the successful utilization of interventions by users and the subsequent sustained performance of the intended health behaviors. This utilization has been generally referred to as “engagement” or “user-engagement.” This term is largely recognizable, but at the same time, abstract and difficult to measure accurately in different settings and contexts. This measurement limitation can be mitigated by applying frameworks/models that guide the way engagement is considered, measured, and applied in analysis within an intervention. Precise definitions and measurements for engagement will allow for better estimation of DBCI effectiveness and provide accurate insights that inform relevant intervention recommendations [4].

### Goal

The aims of this study are (1) to provide an analysis of current engagement definitions and models in the literature, (2) to propose a new model that builds on existing frameworks but addresses current limitations, and (3) to describe the implications for applying the proposed framework to measurements and analysis within DBCI.

### Analysis of Current Definitions of Engagement in Literature

It is generally accepted that user engagement with DBCI is a precursor to improved health outcomes. Growing evidence supports that interaction with DBCI and adherence to intervention features improve the likelihood of reaching desired health outcomes [5]. There is currently no universally accepted and comprehensive definition for engagement, and this has two main implications. First, there is a limited and inconsistent understanding of the engagement’s specific impact, particularly on DBCI effect size, attrition, and sustained health behaviors [4]. Second, there is also a lack of precision in DBCI measurement and evaluation, as the term “engagement” can be interpreted differently across industries (eg, marketing, psychology, and human-computer interaction) [6].

One definition of engagement evaluates esthetics and navigability, particularly how users interface with digital interventions [7]. This type of engagement typically observes usability, measuring interactions with features and functions of the digital solution. This definition is important because it provides insight into the level of use and interaction of a digital intervention. It also informs what components of the intervention users enjoy or use the most and opportunities to leverage these insights to inform future interventions.

Engagement on this level is an important element for the digital intervention scale. O’Brien and Tom [8] defined engagement as an assessment of a user experience (interaction and involvement) with an innovation or technology-based intervention [8]. They argue that this experience leads to the formation of sentiments that impact a user’s likelihood to advocate the use of the innovation among others within their social networks. Furthermore, this definition of engagement helps researchers understand more about the type of users involved in their intervention (ie, data from this engagement addresses questions such as the emerging user-engagement hierarchy identified via subgroups based on user interaction with the features of the digital intervention) [9]. With an understanding of the baseline users of an intervention and their characteristics, researchers can build updates to digital interventions that positively support most types of users [8].

Nevertheless, there are limitations with this type of engagement definition. Definitions based exclusively on user interactions assume that engagement with the digital innovation ultimately leads to the intended intervention outcome (ie, higher engagement and adoption lead to desired health outcomes). This assumption is problematic because of the current limited interpretational challenges user interaction data present, such as the difficulty in distinguishing online versus offline interactions and the implications for intervention exposure, or the absence of frameworks that identify unique meaningful interaction sequences with digital features that translate to specific behavior change techniques (BCTs) embedded in a DBCI [10]. Therefore, this level of engagement definition is insufficient in meeting the need for better and more precise measurement of engagement to inform the effectiveness of DBCI.

Some researchers conceptualize engagement in terms of a user performing the intended health behaviors within an intervention [11]. This type of engagement seeks to understand the relationship between using the digital intervention and behavior change, drawing on evidence-based principles and behavioral theories to evaluate changes in health outcomes [11,12]. The advantage of this definition is that it is ultimately concerned with whether an individual performed the desired health behavior. This definition can closely tie the effectiveness of an intervention to a measure of engagement. Definitions of engagement based on behavior performance parameters only do not provide details of user interactions with components of a digital intervention. Thus, an understanding of what specific digital features are linked with behavior engagement (ie, what features are effective) is absent. This is a significant limitation because data needed to update features of the intervention are absent; thus, future optimization and cost-saving processes informed by evidence will be nearly impossible [13]. Therefore, one is left with a *potentially* effective DBCI and no understanding of what features contributed to their efficacy, thus lacking insights to inform intervention optimization and scale.

The absence of a standardized DBCI framework for defining and measuring engagement inhibits the ability to understand the mechanisms of action of an intervention. Therefore, current approaches are limited in providing accurate and detailed measurement data necessary to demonstrate effectiveness of DBCI.

A proposed model should include definitions of engagement related to DBCI feature interaction as well as performance of the desired health behavior [14]. This approach can inform which specific features of a DBCI influence performance of a health behavior. Essentially, an integrated model should be able to address the relationship between user interaction with the DBCI and the user health behaviors, thus answering the question, “*What level of user-engagement with the DBCI, and by which users, leads to a desired health?*”

Furthermore, this model should ideally delineate and identify unique, meaningful interaction sequences that represent digital features informed by specific behavior change theory–informed components of the intervention (eg, BCTs) [13]. This hierarchical representation of user feature interaction and intervention exposure to BCTs informs a robust dataset that contains explanatory variables that enable a deeper DBCI analysis in real-world settings and an explanation of the mechanism of action with regard to change processes [13]. For this model to be complete, we need to account for behavioral determinants that mediate or moderate the association between digital features engagement and their impact on associated intervention health behavior outcomes. These determinants should include determinants related to user engagement with DBCI [15]. Examples include technology self-efficacy; satisfaction with the DBCI; intervention usability [15]; and technology-associated determinants informed by the technology acceptance model (TAM), such as perceived usefulness, perceived ease of use, and perceived compatibility [16]. In addition, psychosocial determinants rooted in behavioral theories relevant to the behaviors of interest such as the social ecological model [17]; Capability, Opportunity, Motivation - Behavior (COM-B) model [18]; and social cognitive theory [19] must be included in the proposed engagement model. A model housing both engagement definitions (engagement with DBCI and desired health behavior, technology determinants, psychosocial determinants, and outcomes) is robust enough to inform datasets that support rigorous evaluation and measurements of DBCI.

## The Johnson and Johnson Approach to Health Engagement: Definitions and Framework

### Overview

Following the abovementioned recommendation outlining a proposed model for defining and measuring engagement, we present a definition and model for engagement. Several steps were taken to achieve this goal.

First, several definitions of engagement were reviewed from the literature and practice-based knowledge surrounding DBCI [7,11-15]. These definitions were analyzed to identify current limitations.

Second, new definitions for engagement were proposed. The intention here was to have new sets of definitions that addressed the observed limitations of current definitions and to standardize terminologies around DBCI engagement with the intention of creating better DBCI measurements.

### Defining “Big E” and “Little e” Engagement

“Big E” is engagement with the targeted health behavior, hereafter referred to as health behavior engagement, and is the primary outcome of a DBCI. However, the goal is to attain health outcomes through the health behavior. Thus, it is important that engagement with the health behavior is achievable and measurable to determine if a DBCI is successful.

“Little e” is engagement with the digital behavior change intervention. This is sometimes referred to as user interaction with the digital solution. Hereafter, the term DBCI engagement will be used to represent engagement with the digital solution. DBCI engagement is comprised of two types of interactions:

- User interactions with the DBCI features and the context in which those interactions happen (little e_UI_)
- Interactions with behavior change intervention components/active ingredients specifically designed to influence the behavioral determinants which in turn influence the health behaviors (little e_BCT_)

**Figure 1:**
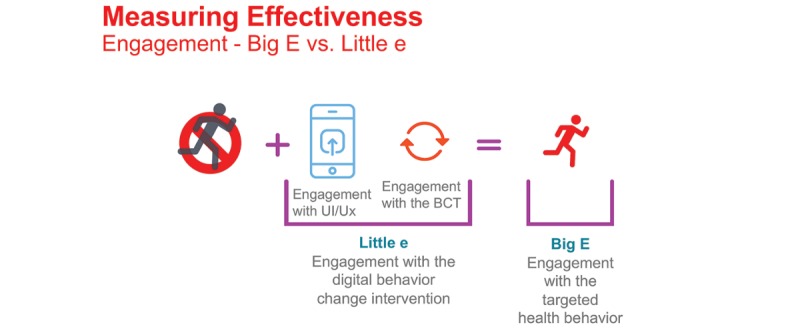
Diagram illustrating the Johnson and Johnson approach to health engagement. UI: user interaction; Ux: user experience; BCT: behavior change technique.

The success of health behavior engagement is dependent upon DBCI engagement. If users do not interact with DBCI features, exposure to the behavior change intervention components will be less likely to influence behavioral determinants and lead to health behavior engagement across a broad population. Health behavior engagement is also dependent upon the quality and relevance of the behavior change intervention components within the solution. Therefore, user interactions and behavior change intervention components combine to create Little e; this in turn influences Big E. Figure 1 illustrates how these definitions of engagement inform the overall intervention measurement.

**Figure 2:**
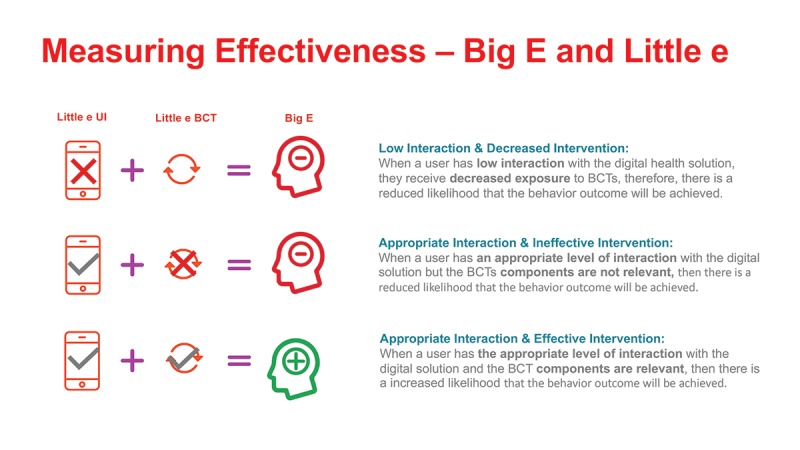
Diagram illustrating relationship engagement measurements. UI: user interaction; BCT: behavior change technique.

Figure 1 indicates that a sedentary individual will need to interact with the appropriate level of the DBCI features (little e_UI_) to be exposed to the right and effective level of behavior change intervention components (little e_BCT_). This should ideally lead to changes in determinants that support engagement with the health behavior. Inadequate levels of little e_UI_ interaction or ineffective exposure to appropriate BCTs will not lead to health behavior engagement (Figure 2). In some cases, appropriate exposure to a BCT is based on what is clinically relevant to influence a health behavior and by extension, a health outcome. For example, physical activity (steps) tracking is a BCT (self-monitoring of a behavior), but attaining *x* number of steps will be needed to begin seeing a decrease in *y* amount of weight (health outcome).

Principles from program planning and intervention design literature [20] were leveraged to develop a framework model unique for building DBCI. This model is a hybrid that uses the layout of a theory of change conceptual model and a traditional logic model (Table 1) to provide the structure upon which we systematically embedded the new engagement definitions and then tested the robustness of these definitions in order to produce the right amount of data to inform complete and accurate measurements of DBCI effectiveness.

**Table 1:** The Johnson and Johnson approach to health proposed engagement definitions and framework. As with a traditional logic model, this table should be read from left to right. The underlying assumption is that exposure to an intervention should lead to changes in determinants that in turn influences health behaviors, health outcomes, and organizational outcomes. For each phase of the logic model, the table illustrates examples of the engagement category, measurement category, sample metrics, and the metrics/data source.

Logic model category	Exposure to intervention		Determinants	Health behavior	Health outcomes	Organizational outcomes
Engagement category	Little e engagement with the DBCI^a^		—^b^	Big E engagement in health behaviors	Outcome	Outcome
Measurement category	UI^c^ interactions Contextual information	BCT^d^ intervention components	Capability Opportunity motivation	Health behaviors	Health outcomes at individual level	Organizational outcomes
Sample metrics	Clicks Swipes Location data	Goal setting Restructuring thoughts	Change in: Skills Self-efficacy to perform physical activity Access to food store with health meal options	Sufficient level of physical activity Targeted nutrition behaviors	Improved A1C Maintain ideal weight	Descrease demand of health care system
Metric/data source	In-app	In-app	In-app/out of app	In-app/out of app	In-app/out of app	In-app/out of app

^a^DBCI: digital behavior change interaction.

^b^Not applicable.

^c^UI: user interface.

^d^BCT: behavior change technique.

This framework provides an engagement category that details how features of Little e inform Big E and builds upon components that align with an intervention logic model, such as exposure to the intervention, determinants, health behaviors, health outcomes, and organizational outcomes. There were five steps that informed the process of building the model and its components:

1. Utilization of market research, economic evaluation, and subject matter expertise in therapeutic areas to inform our choice of the organizational, individual health, and behavior outcomes (ie, we knew what specific behaviors needed to be performed to lead to the outcomes of interest and by what effect size).
2. Review of the behaviors and created subbehaviors and specific performance indicators associated with them.
3. Examination of the behavior science literature to deduce the factors (mediator or moderators) that influence the performance of the behaviors among individuals in the target population. These factors were grouped using various frameworks such as (a) enabler and barriers; (b) mediators or moderators; (c) factors personally and environmentally informed by the socioecological model [17]; and (d) capability, motivational, or opportunity factors [18].
4. Use of the understanding and learnings from several theories, such as the classical and operant processes, social cognitive theory, social learning theory, and self-determination theory [19,21-23], to determine which behaviors fall within the involuntary and voluntary process. Thereafter, we used this guidance to determine antecedent and consequence behavioral determinants (ie, BCTs that make up components of the intervention).
5. Use of the understanding from the intervention mapping literature [20] to determine specific change processes that illustrated our best-informed assumptions about which specific BCTs drive factors influence performance of the behaviors. This helped define and guide the digital product strategy where each behavior change process is represented by a set of user experience/user interaction (UI) and content features.

The measurement category provides additional detail of what factors should be measured in each engagement category. Finally, the example metric category provides examples of metrics used during evaluation.

## Implications of the Engagement Framework for Interventions Measurements

### Overview

The framework provides a structure to discuss implications for the measurement and analysis of DBCI. These implications will be discussed under the categories of the engagement framework. They include exposure to the intervention, determinants, behavior, and outcomes. These categories are organized in a manner that provides information on the potential causal pathway of change between intervention exposure and outcomes, that is, exposure to the intervention (Little e) influences determinants that influences behaviors and ultimately leads to health outcomes. Insights on approaches to DCBI analysis using engagement data will also be discussed in this paper.

### Exposure to the Intervention (Little e)

This category is comprised of two engagement types: (1) user interaction with the DBCI features (little e_UI_) and (2) interactions with the behavior change interventions components (little e_BCT_).

### User Interaction With the Digital Behavior Change Interventions

This refers to user interaction with the technology. Exemplary categories of digital solution interactions that could be measured include number of logins, clicks, swipes, time spent interacting with each feature, order of interactions (path taken), and real-time feedback/assessment of user experience. These interactions can be captured within the solution at the backend by using combinations of product analytics data, representing user-level individual interactions data over time (ie, longitudinally). It is critical to collect individual level, time-series data and specify this request upon the development of a DBCI solution. Often, however, out-of-box analytical programs do not have this capability as the default setting.

### Interactions With Behavior Change Intervention Components

Interaction with, and subsequent exposure to, the behavior change intervention components (little e_BCT_) is designed specifically to influence the determinants of behavior [15]. Using theory-informed models [17,19-23], we can detail which user little e_BCT_ interactions are associated with specific health behaviors. In traditional intervention design, these are the key components of behavior change theory that are used to influence changes in determinants, and ultimately, health behaviors.

**Figure 3:**
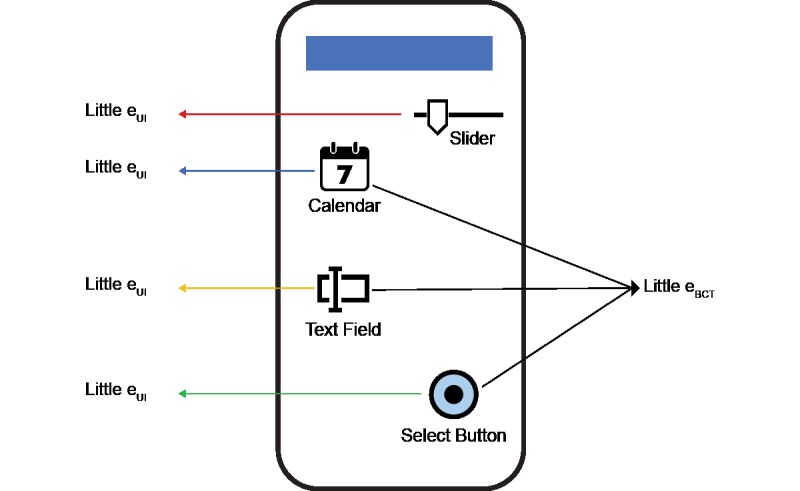
Diagram illustrating unique constellation of user interaction/user interface features that represent behavior change techniques. UI: user interaction; BCT: behavior change technique.

Behavior change interventions are most effective when developed using behavior change theory [2]. Therefore, we have to understand exposure to behavior change techniques to determine specifically which behavior change intervention components are important to design and then measure in the DBCI. Behavior change techniques are the smallest component parts of widely used theories of health behavior, sourced from various fields and through consensus of experts [10]. BCTs are used because they enable researchers to focus on specific components of an intervention in order to determine how much each aspect of the intervention is contributing to the desired outcome. In other words, use of behavior change techniques, the smallest component part of a behavior change theory, allows us to parse out the effect of each behavior change technique, thus developing an understanding of which technique works for which population and outcome. When these insights are developed in a real-world setting across a range of users, they can be translated for a larger group of users to obtain better outcomes. These BCTs are often experienced as features in the user experiences (Figure 3) and, in some cases, inform how the technology solutions may function.

Further, BCTs arranged in a taxonomy, or ontology, allow for organization of the components in technology design by making behavior change theory concepts machine readable (eg, hierarchically categorized and mutually exclusive), which enables behavior science to converge with data science [24]. Examples of BCTs include goal setting, action planning, feedback, monitoring, and problem solving. A full list of behavior change techniques commonly used in various fields of behavior science can be found in The Behavior Change Technique Taxonomy (v1) of 93 Hierarchically Clustered Techniques developed by Michie et al [10]. This approach enables the identification of active ingredients of the DBCI (ie, the behavior change intervention components that are most critical to improve behavior) and ultimately determine a dose-response relationship between the DBCI and the desired health behavior and health outcome.

Measurement of behavior change intervention components (little e_BCT_) can be represented by a combination of user interface interactions. Some of the same interactions captured for understanding user interaction with the system (little e_UI_) can be combined to create measures for DBCI components/active ingredients (Figure 3).

In this illustration, the app user is exposed to an “Action Planning” BCT (little e_BCT_) via a combination of calendar text features and a select button (little e_UI_) to schedule an exercise routine.

### Determinants

Determinants refers to the influence of factors that shape individual decisions and actual performance of the behaviors. Prior work on digital health technology engagement models advocate for examination of two main level of determinants: (1) determinants of user engagement with the intervention (eg, DBCI) and (2) psychosocial determinants [15]. Determinants of user engagement focus on the influences that support user interaction with a DBCI. For example, technology self-efficacy, satisfaction with the DBCI, intervention usability, and navigability and technology perceived compatibility and ease of use [15,16]. Psychosocial determinants refer to influence of factors (usually social) on individual/group decision and performance of behaviors (desirable or not) [25]. These factors are informed by various behavior science theories such as the COM-B model, socioecological model, the classical and operant processes, social cognitive theory/social learning theory, and self-determination theory [17-19,22,23].

Both types of determinants can be grouped as mediators or moderators and help tie both definitions of engagement (Big E and Little e) together. According to the COM-B model, behavior change occurs when motivation meets capability and opportunity. Specifically, for a behavior to happen, the person must have the capability, opportunity, and motivation to perform the behavior [18]. In our Johnson and Johnson approach to health engagement framework, behavior is defined as an observable action that a person does, capability is a physical and psychological ability to perform the behavior, opportunity is an immediate environmental factor(s) that promotes or enables a behavior, and motivation refers to the mental resolve to direct a behavior [18].

Within the DBCI, determinants can be captured in app through assessments, although the measurement of determinant is not critical. These assessments could be periodic or based on a predefined logic (for example, business rules informing a DBCI). In some instances, this predefined logic is built around specific ecological and environment contexts that are timed or random. These types of assessments are generally referred to as an ecological momentary assessment (EMA) [26]. In the context of a study or research, determinants could be measured using a survey. Determinants are important to measure the effectiveness of intervention components on behavior and outcomes. Therefore, it is important to ensure they can be measured within DBCI during product testing to understand the full mechanism of action. Once a DBCI is released, testing of determinants is unnecessary if no new audiences or population subgroups are introduced. When choosing a data capture method, it is critical to weigh the value of the data to be obtained with user burden expected. This is because a tedious user experience on data capture (eg, more burden on the user to actively track data) can, in turn, influence user engagement determinants negatively [27].

### Health Behavior (Big E)

In this model, we categorize health behaviors as lifestyle behaviors, health care–related behaviors, and one-off behaviors. Lifestyle behaviors refer to activities performed very frequently, such as physical activity, sleeping, and eating. Examples of health care–related behaviors include adherence to medication and treatment plans, while getting a flu shot is an example of a one-off health behavior. Health behaviors can be measured though a variety of ways (in app, EMAs, surveys, etc). However, when possible, behaviors should be captured passively using in-app or out-of-app sensors to ensure accuracy of the data and reduce user burden on the people to track and monitor their behavior (except in cases when the intervention specifically calls for it).

## Analysis of Digital Behavior Change Interventions Using Engagement Data

### Overview

Data from the engagement framework ultimately aims to describe specific relations between Little e and Big E and explains the pathway of these relationships through the influence of determinants. In other words, DBCI analysis seeks to understand which BCT exposures lead to which health behavior and through the effects of which determinants. This framework informs the development of a specific dataset that addresses these questions. There are two broad approaches to consider when analyzing such data: an a priori theory approach and a grounded theory approach.

### A Priori Theory Approach

In this approach, DBCI interactions and health behavior engagement can be identified or assigned as the solution has been designed. This is largely informed by theory and learnings from behavior-based intervention implementation literature, such as intervention mapping [20]. A priori definition helps determine if specific assumptions or theoretical concepts utilized in the design of the DBCI solution hold true. For example, if a DBCI feature was designed to help users address a specific motivational determinant of behavior, and the feature was never utilized, you would not expect the motivational determinant to be influenced through that feature/conceptual approach. Similarly, if the feature was utilized, but the person’s motivation did not change, you may question whether this component was an effective way to address this motivational factor. The critical limitation of this approach, however, is that researchers are restricted to the number of behavior change pathways (ie, from Little e to Big E) that were originally accounted for. Emerging new change processes could be potentially missed if they were not initially built in (ie, the analysis of the intervention is only as good as the change processes and pathways originally captured).

### A Grounded Theory Approach

In this approach, DBCI interactions and health behaviors are interpreted in real time or after the solution has been used to develop new learnings in order to make the DCBI more effective, if needed. In this instance, methods such as cluster analysis or path analysis can help understand how users experience the intervention, identify useful features, and determine various user pattern groups (that might indicate which path is most successful for specific types of users) [28,29]. This approach helps identify patterns and interactions that the user takes. In some cases, the emerging user engagement patterns and change processes were not intuitive or originally intended. These findings can be used for further exploration and to inform other types of analyses. However, there could be a challenge in capturing and interpreting the data and thus running the risk of spurious associations and findings if they are not vetted by evidence or do not fit the schema of an existing theory [28].

In summary, there is potential value in each approach. However, the pros and cons of both approaches should be carefully considered when making analytic decisions.

## Conclusion and the Promise of Engagement Data to Inform Measurement of Digital Behavior Change Interventions

Measurement of both little e_UI_ and intervention BCT components (little e_BCT_) are necessary to understand the effectiveness of DBCI on Big E (health behavior). DBCI guided by the Johnson and Johnson approach to health engagement framework helps clarify exactly which actions a user takes (path) and the context in which such interaction and health behavior occur. Due to advancements in technology, it is possible to collect data over time regarding Little e and Big E and to consider additional determinants’ data on dynamic contextual information (eg, location, time of day, and biometric characteristics like heart rate). Other determinant information (including demographics) are also important and should be leveraged to build user profiles that are robust and describe behavior change processes from Little e to Big E and influence our understanding of behavior change theories [15].

The full potential of digital health solutions can be achieved when insights from human behaviors can be observed and measured discretely to enhance and improve our existing models of behavior change theory, which were developed mostly using static measures of behaviors. Given the power of technology, we are now able to capture fluid behaviors of an individual and are better equipped to address the question of which intervention works best for whom and for what outcome.

It is also possible to measure mechanisms that influence adoption and maintenance of health behaviors to build product efficiencies and effectiveness (relevant to the greatest audience subgroup as feasible). Such mechanisms include Little e (interaction with UI + interaction with BCT components=health behavior via a determinant). Equipped with data about user interactions with technology, exposure to intervention BCT components, and execution of health behavior, we can answer questions of not only *if* the intervention, but also *how* the intervention worked and for *whom*.

We can identify and capitalize on efficiencies and observed insights to optimize a user’s path to the health behavior and increase the likelihood of sustaining that health behavior. Evidence has shown that tailored and individualized interventions are successful for improving health behavior and health outcomes [30]. Utilizing all data available (little e_UI_, little e_BCT_, Big E), statistical and computational analyses can be utilized to understand patterns of human behavior, identify determinants of human behavior, and personalize interventions specifically for the person receiving the intervention [29,30]. By studying little e_UI_, little e_BCT_, and health behaviors in tandem and separately, we can address questions such as the following:

- What is the best user experience for users with particular characteristics?
- What is (are) the most appropriate set of behavior change technique(s)/intervention components, given a user has a certain set of characteristics?
- What is the next appropriate intervention component to provide a user, given they have had positive technology interactions with these components previously?

Essentially, through these data, we become equipped to tailor and personalize interventions without requiring a static battery of psychosocial and preference questions in the initial interaction.

Finally, to reach the full potential of technology applied to behavior change (DBCI), there is a need to draw from diverse populations and observe and learn how DBCI works for different people. Implementing an engagement framework–guided DBCI at scale enables the gathering of a diverse set of data while simultaneously diversifying the intervention appropriately at scale, rather than starting in small pockets and building health technologies that are inflexible.

## Abbreviations

BCT: behavior change technique
COM-B: Capability, Opportunity, Motivation - Behavior
DBCI: digital behavior change intervention
EMA: ecological momentary assessment
TAM: technology acceptance model
UI: user interaction
